# Systematic improvement of isobutanol production from d-xylose in engineered *Saccharomyces cerevisiae*

**DOI:** 10.1186/s13568-019-0885-3

**Published:** 2019-10-10

**Authors:** Peerada Promdonkoy, Wiparat Siripong, Joe James Downes, Sutipa Tanapongpipat, Weerawat Runguphan

**Affiliations:** 1 National Center for Genetic Engineering and Biotechnology, 113 Thailand Science Park, Paholyothin Road, Klong 1, Klong Luang, Pathumthani, 12120 Thailand; 2 University of Kent, Canterbury, Kent UK; 3 Present Address: Syngenta, Jealott’s Hill International Research Station, Bracknell, Berkshire RG42 6EY UK

**Keywords:** Yeast, Xylose utilization, Advanced biofuel, Isobutanol, Metabolic engineering

## Abstract

As the importance of reducing carbon emissions as a means to limit the serious effects of global climate change becomes apparent, synthetic biologists and metabolic engineers are looking to develop renewable sources for transportation fuels and petroleum-derived chemicals. In recent years, microbial production of high-energy fuels has emerged as an attractive alternative to the traditional production of transportation fuels. In particular, the Baker’s yeast *Saccharomyces cerevisiae*, a highly versatile microbial chassis, has been engineered to produce a wide array of biofuels. Nevertheless, a key limitation of *S. cerevisiae* is its inability to utilize xylose, the second most abundant sugar in lignocellulosic biomass, for both growth and chemical production. Therefore, the development of a robust *S. cerevisiae* strain that is able to use xylose is of great importance. Here, we engineered *S. cerevisiae* to efficiently utilize xylose as a carbon source and produce the advanced biofuel isobutanol. Specifically, we screened xylose reductase (XR) and xylose dehydrogenase (XDH) variants from different xylose-metabolizing yeast strains to identify the XR–XDH combination with the highest activity. Overexpression of the selected XR–XDH variants, a xylose-specific sugar transporter, xylulokinase, and isobutanol pathway enzymes in conjunction with the deletions of *PHO13* and *GRE3* resulted in an engineered strain that is capable of producing isobutanol at a titer of 48.4 ± 2.0 mg/L (yield of 7.0 mg/g d-xylose). This is a 36-fold increase from the previous report by Brat and Boles and, to our knowledge, is the highest isobutanol yield from d-xylose in a microbial system. We hope that our work will set the stage for an economic route for the production of advanced biofuel isobutanol and enable efficient utilization of lignocellulosic biomass.

## Introduction

Renewable fuels have received significant interest in recent years due to environmental concerns and unsustainable energy demands (Peralta-Yahya et al. 2012; Liao et al. 2016). Microbial production of advanced fuels has emerged as a viable alternative to the traditional petroleum-based transportation fuels that are responsible for the emission of greenhouse gases (Flores et al. 2019). Recent examples include the production of long chain alkanes and fatty acid ethyl esters (FAEE) as diesel replacements and short-chain alkanes and isoprenoid-based biofuels as gasoline replacements (Peralta-Yahya et al. 2011; Choi and Lee 2013; Runguphan and Keasling 2014; Buijs et al. 2015). In many of these examples, the yeast *S. cerevisiae* has been the host of choice due to its ideal properties, namely: (1) high tolerance to many industrial stresses, such as low pH and high osmotic pressure; (2) high tolerance to the desired biofuel products; (3) resistance to phage infection; and (4) availability of genetic tools and “omics” data for bottoms-up strain engineering. However, despite its potential as a production host, *S. cerevisiae* suffers from one significant drawback: it is unable to utilize xylose, a C5 sugar that is a major component (~ 30–40%) of lignocellulosic biomass (Kwak and Jin 2017).

Increasingly, the use of starch or the sucrose fractions of agricultural crops as the carbon substrates for biomass conversion has become less attractive due to environmental, economical and ethical considerations (Mosier et al. 2005). Instead, lignocellulosic biomass, such as agricultural residues and energy crops, offers a cheap and renewable alternative carbon source for the microbial production of biofuels. Since xylose is the second most abundant component of lignocellulosic biomass, enabling the yeast *S. cerevisiae* to efficiently utilize xylose and convert it into biofuels is of great importance and has been the focus of intense and highly competitive research (Li et al. 2019).

**Fig. 1:**
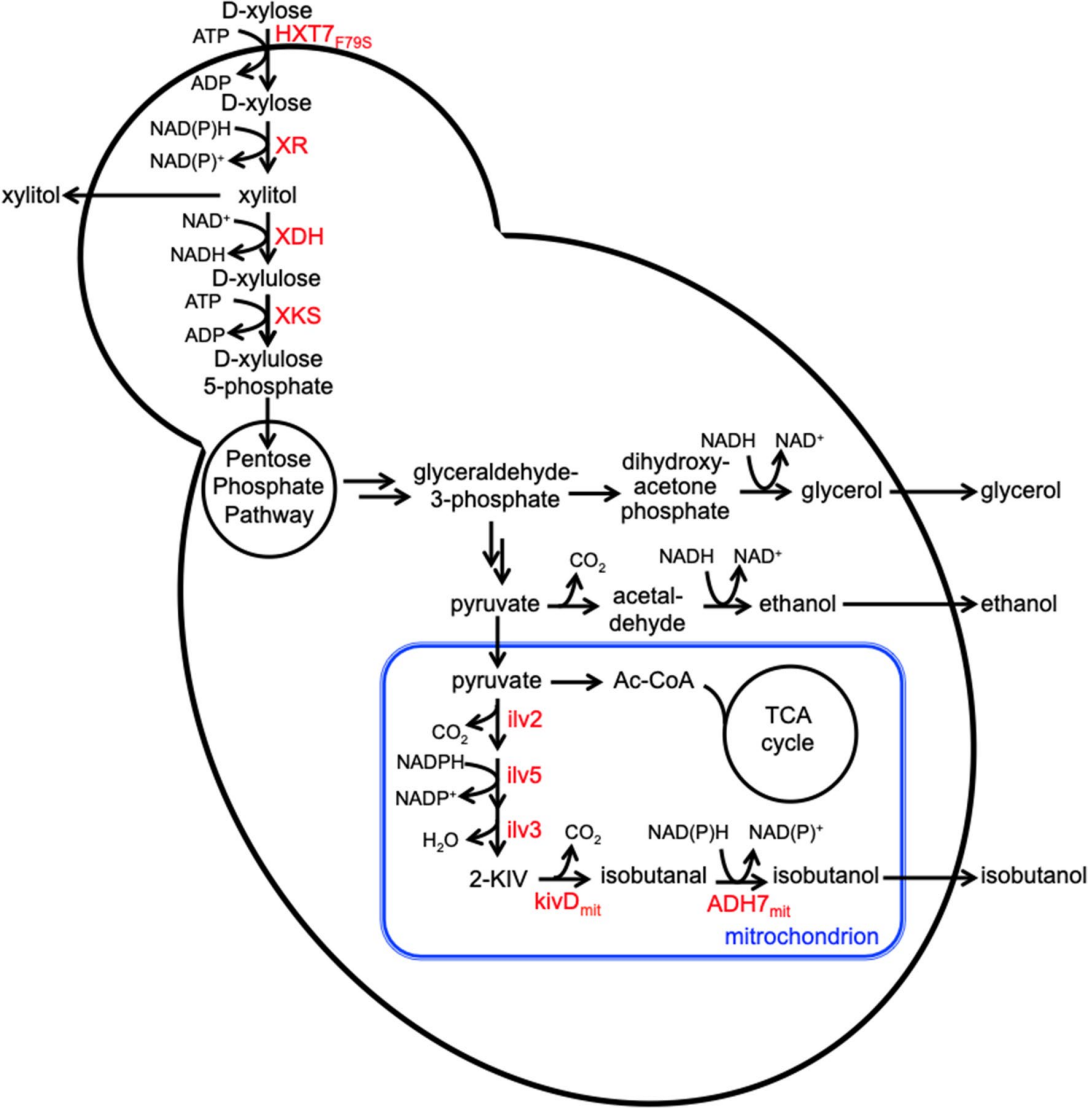
Engineered production of isobutanol from d-xylose in *S. cerevisiae. HXT7*_*F79S*_ high affinity glucose transporter with a F79S point mutation, *XR* xylose reductase, *XDH* xylitol dehydrogenase, *XKS* xylulokinase, *Ilv2* acetolactate synthase, *Ilv5* acetohydroxyacid reductoisomerase, *Ilv3* dihydroxyacid dehydratase, *kivD*_*mit*_ mitochondria-targeted keto-acid decarboxylase, *ADH7*_*mit*_ mitochondria-targeted alcohol dehydrogenase. Enzymes highlighted in red are overexpressed

An extensive body of work has resulted in engineered strains that are capable of producing near-theoretical yields of ethanol from pure xylose and xylose-containing biomass (Zhang et al. 2016). To create such a strain, a xylose assimilation pathway is introduced into *S. cerevisiae*, enabling the resulting strain to convert xylose into xylulose and then xylulose-5-phosphate, a starting metabolite to the non-oxidative Pentose Phosphate Pathway (PPP) (Fig. 1) (Moyses et al. 2016). The two products of the PPP, glyceraldehyde-3-phosphate and fructose-6-phosphate, then enter glycolysis, producing pyruvate and leading to ethanol production.

Two distinct pathways exist in xylose-fermenting organisms for the conversion of xylose to xylulose: the xylose reductase–xylitol dehydrogenase (XR–XDH) pathway in yeast and aerobic fungal species and the xylose isomerase (XI) pathway in bacteria and anaerobic fungal. In the XR–XDH pathway, XR first reduces xylose to xylitol using NADPH and/or NADH as a cofactor. XDH then converts xylitol into xylulose with a specific cofactor preference for NAD^+^. Because of the dual cofactor preference, the redox imbalance hinders successful implementation of the XR–XDH pathway. Conversely, in the XI pathway, XI converts xylose to xylulose without requiring any cofactor. However, as XI enzymes tend to have lower catalytic efficiency compared to XR–XDH enzymes, they are less attractive for industrial-scale applications (Kwak and Jin 2017).

Although microbial ethanol fermentation has played an important role in the transition from conventional fossil fuels to bio-based fuels, ethanol is not an ideal gasoline replacement due to its poor physicochemical properties (Generoso et al. 2015). Specifically, ethanol has low energy density (~ 70% of the energy content of gasoline), high tendency to absorb water, and high vapor pressure. In contrast, higher and branched-chain alcohols, such as butanol and isobutanol, have superior energy density and are more compatible with the existing distribution and storage infrastructures (Lamsen and Atsumi 2012). Moreover, branched-chain alcohols have higher octane numbers compared with their straight-chain counterparts and are therefore ideal gasoline substitutes for high performance petrol engines.

To produce branched-chain and higher alcohols in a non-native producer, Atsumi and coworkers devised a strategy which involves “hijacking” the native amino acid metabolism of the host organism and diverts the amino acid intermediate to the 2-keto acid degradation pathway for higher alcohol production (Atsumi et al. 2008). In the l-valine biosynthetic pathway, acetolactate synthase (Ilv2) condenses two molecules of pyruvate to 2-acetolactate, which is then reduced to 2,3-dihydroxyisovalerate by acetohydroxyacid reductoisomerase (Ilv5). Next, 2,3-dihydroxyisovalerate is converted to 2-ketoisovalerate, a key intermediate in l-valine biosynthesis, by dihydroxyacid dehydratase (Ilv3). In the ketoacid degradation pathway, 2-ketoisovalerate is converted to isobutanal by introducing a heterologous ketoacid decarboxylase (kivD) from the bacterium *Lactococcus lactis* or by overexpression of the endogenous decarboxylase (Aro10). The resulting aldehyde is then reduced by an alcohol dehydrogenase (Adh7 or Adh2) from *S. cerevisiae* to the final alcohol products, including isobutanol. Though the original work was done in *E. coli*, several labs have since applied this strategy to produce isobutanol in many organisms including the cyanobacterium *Synechococcus elongatus* PCC7942, the soil bacterium *Ralstonia eutropha*, and more recently, the methylotrophic yeast *Pichia pastoris* (Atsumi et al. 2009; Lu et al. 2012; Siripong et al. 2018).

While production of isobutanol from glucose in the yeast system is well established, we found only one previous report of branched-chain alcohol production from xylose in *S. cerevisiae* (Brat and Boles 2013). In the study, Brat and Boles overexpressed the xylose isomerase XylA from *Clostridium phytofermentans* along with isobutanol pathway enzymes in *S. cerevisiae*. The engineered strain produced isobutanol at a yield of 0.16 mg/g xylose, well below the reported yields from glucose of up to 16 mg/g glucose (Matsuda et al. 2013).

Here, we improved upon this previous work and engineered an efficient isobutanol producing strain of *S. cerevisiae*. Specifically, we first screened XR–XDH pathway genes from various xylose-utilizing species to identify an optimal XR–XDH gene combination. We then integrated the isobutanol pathway, which comprises the first portion of the endogenous l-valine biosynthesis and keto-acid degradation pathway. Next, we disrupted two endogenous genes that have previously been shown to improve xylose assimilation in *S. cerevisiae*. Finally, we fine-tuned the expression level of the each gene in the xylose utilization pathway and isobutanol pathway. We envision that our yeast platform will pave the way for a scalable and economic route to the production of biofuels utilizing environmentally sensitive and less carbon-intensive agricultural wastes and energy crops.

## Methods

### Yeast strain, media and transformation

The yeast strains used in this study were constructed from BY4742 [derivative of S288C (*Mat α; his3Δ1; leu2Δ0; lys2Δ0; ura3Δ0*)] (Table 1). The plasmids used in this study were generated from the pRS416, pRSII416 and pRSII426 vector series (Additional file 1: Table S1) (Chee and Haase 2012). pRS416 and pRSII416 contain the yeast CEN6/ARS4 origin of replication, which results in transformants with a low copy plasmid number (1 copy per cell). In contrast, pRSII426 contains the yeast 2 μ origin of replication, which allows autonomous replication of the plasmids and results in transformants with a high plasmid copy number (10–40 copies per cell) (Schneider and Guarente 1991).

**Table 1:** Strains generated in this study

Strain name	Genotype	Description	References
BY4742	*Mat α; his3Δ1; leu2Δ0; lys2Δ0; ura3Δ0*	Laboratory strain	Baker Brachmann et al. (1998)
PWY0013	BY4742 *ARS208::P*_*TEF1*_-*SsXKS; YMRWΔ15::P*_*TEF1*_-*HXTF79S*	BY4742 overexpressing *XKS* from *Scheffersomyces stipitis*, *HXT7*_F79S from *Saccharomyces cerevisiae*	This study
PWY1113	PWY0013 *ARS308::P*_*TEF1*_-*CtXYL1; ARS720::P*_*TEF1*_-*CtXYL2*	PWY0013 overexpressing *XYL1* and *XYL2* from *Candida tropicalis*	This study
PWY1213	PWY0013 *ARS308::P*_*TEF1*_-*CsXYL1; ARS720::P*_*TEF1*_-*CtXYL2*	PWY0013 overexpressing *XYL1* from *Candida shehatae* and *XYL2* from *C. tropicalis*	This study
PWY1313	PWY0013 *ARS308::P*_*TEF1*_-*SsXYL1; ARS720::P*_*TEF1*_-*CtXYL2*	PWY0013 overexpressing *XYL1* from *S. stipitis* and *XYL2* from *C. tropicalis*	This study
PWY1413	PWY0013 *ARS308::P*_*TEF1*_-*SpXYL1.1; ARS720::P*_*TEF1*_-*CtXYL2*	PWY0013 overexpressing *XYL1.1* from *Spathaspora passalidarum* and *XYL2* from *C. tropicalis*	This study
PWY1513	PWY0013*; ARS308::P*_*TEF1*_-*SpXYL1.2; ARS720::P*_*TEF1*_-*CtXYL2*	PWY0013 overexpressing *XYL1.2* from *S. passalidarum* and *XYL2* from *C. tropicalis*	This study
PWY2113	PWY0013 *ARS308::P*_*TEF1*_-*CtXYL1; ARS720::P*_*TEF1*-_*SsXYL2*	PWY0013 overexpressing *XYL1* from *C. tropicalis* and *XYL2* from *S. stipitis*	This study
PWY2213	PWY0013 *ARS308::P*_*TEF1*_-*CsXYL1; ARS720::P*_*TEF1*_-*SsXYL2*	PWY0013 overexpressing *XYL1* from *C. shehatae* and *XYL2* from *S. stipitis*	This study
PWY2313	PWY0013 *ARS308::P*_*TEF1*_-*SsXYL1; ARS720::P*_*TEF1*_-*SsXYL2*	PWY0013 overexpressing *XYL1* and *XYL2* from *S. stipitis*	This study
PWY2413	PWY0013 *ARS308::P*_*TEF1*_-*SpXYL1.1; ARS720::P*_*TEF1*_-*SsXYL2*	PWY0013 overexpressing *XYL1.1* from *S. passalidarum* and *XYL2* from *S. stipitis*	This study
PWY2513	PWY0013 *ARS308::P*_*TEF1*_-*SpXYL1.2; ARS720::P*_*TEF1*_-*SsXYL2*	PWY0013 overexpressing *XYL1.2* from *S. passalidarum* and *XYL2* from *S. stipitis*	This study
PWY3113	PWY0013 *ARS308::P*_*TEF1*_-*CtXYL1; ARS720::P*_*TEF1*_-*SpXYL2*	PWY0013 overexpressing *XYL1* from *C. tropicalis* and *XYL2* from S*. passalidarum*	This study
PWY3313	PWY0013 *ARS308::P*_*TEF1*_-*SsXYL1; ARS720::P*_*TEF1*_-*SpXYL2*	PWY0013 overexpressing *XYL1* from *S. stipitis* and *XYL2* from *S. passalidarum*	This study
PWY3413	PWY0013 *ARS308::PTEF1*-*SpXYL1.1; ARS720::PTEF1*-*SpXYL2*	PWY0013 overexpressing *XYL1.1* and *XYL2* from *S. passalidarum*	This study
PWY3513	PWY0013 *ARS308::P*_*TEF1*_-*SpXYL1.2; ARS720::P*_*TEF1*_-*SpXYL2*	PWY0013 overexpressing *XYL1.2* and *XYL2* from *S. passalidarum*	This study
PWY1123	PWY1113 *GRE3Δ*	PWY1113 with *GRE3* deleted	This study
PWY1223	PWY1213 *GRE3Δ*	PWY1213 with *GRE3* deleted	This study
PWY2323	PWY2313 *GRE3Δ*	PWY2313 with *GRE3* deleted	This study
PWY3323	PWY3313 *GRE3Δ*	PWY2313 with *GRE3* deleted	This study
PWY1133	PWY1123 *YORWΔ22::P*_*TDH3*_ –*LlkivDmt*-*T2A*-*ScADH7mt*	PWY1123 overexpressing *ADH7* from *S. cerevisiae*, and *kivD* from *L. lactis*	This study
PWY1143	PWY1133 *ARS1309::P*_*TEF1*_-*ScIlv2*-*T2A*-*ScIlv5*-*T2A*-*ScIlv3*	PWY1133 overexpressing *Ilv2, Ilv5, Ilv3* from *S. cerevisiae*	This study
PWY1153	PWY1143 *PHO13Δ*	PWY1143 with *PHO13* deleted	This study
PWY2333	PWY2323 *YORWΔ22::PTDH3* –*LlkivDmt*-*T2A*-*ScADH7mt*	PWY2323 overexpressing *ADH7* from *S. cerevisiae*, and *kivD* from *L. lactis*	This study
PWY2343	PWY2333 *ARS1309::P*_*TEF1*_-*ScIlv2*-*T2A*-*ScIlv5*-*T2A*-*ScIlv3*	PWY2333 overexpressing *Ilv2, Ilv5, Ilv3* from *S. cerevisiae*	This study
PWY2353	PWY2343 *PHO13Δ*	PWY2343 with *PHO13* deleted	This paper

Yeast and bacterial strains were stored in 25% glycerol at − 80 °C. *E. coli* was grown in Luria–Bertani medium. Ampicillin at 100 μg/mL was added to the medium when required. Yeast strain without plasmid was cultivated in YPD medium (10 g/L yeast extract, 20 g/L Bacto Peptone and 20 g/L glucose). Selection of yeast transformants with URA3 was done on a yeast selective medium (6.7 g/L of Yeast Nitrogen Base (Difco), 20 g/L glucose, and a mixture of appropriate nucleotide bases and amino acids with Uracil dropouts (CSM-URA). Yeast cells were cultivated at 30 °C in 50 mL Falcon tubes or 250 mL Erlenmeyer flasks closed with cotton plug and shaken at 250 rpm.

XR and XH candidate genes were amplified from genomic DNA [in the cases where the strains are available in the Thailand Bioresource Research Center (TBRC)] or synthesized by Genscript. The individual genes were placed behind the P_TEF1_ promoter and separately integrated into the yeast genome at specific targets (Reider Apel et al. 2017). Gene knockouts were generated using a previously reported gene disruption cassette in *S. cerevisiae* (Gueldener et al. 2002). Gene disruption cassettes containing the URA3 selectable marker flanked by loxP sites (obtained by PCR of the pUG72 plasmid) were produced with 42 base pairs of homology on either side of each target integration site. Oligonucleotide primers used for PCR, cloning, chromosomal integration and gene knock-outs in this study are included in Additional file 1. Yeast cells were transformed using the Li/Ac/PEG method as previously described (Gietz and Schiestl 2007a, b). Following yeast transformations, colonies were selected on selective medium lacking uracil and confirmed via PCR. The marker gene (URA3) was removed by overexpressing the Cre recombinase to excise the selection marker between the loxP sites in the disruption cassette. This enables subsequent rounds of genomic integrations. Cre recombinase was expressed using the inducible GAL1 promoter on plasmid pSH62 (Hegemann and Heick 2011). The strain harboring pSH62 was grown in SD medium plus 1 g/L 5-fluoroorotic acid to encourage loss of the URA3 (Boeke et al. 1984). To verify the genetic stability of the engineered strains, their genomic DNA was isolated (Promega Wizard Genomic DNA Purification kit) and then subjected to a diagnostic PCR amplification that amplified regions both upstream and downstream of the integration/deletion sites.

### Plasmid construction

*Plasmid pRS416Tef1*-*SsXKS*: XKS (accession number: XM_001387288) was amplified from *S. stipitis* BCC47637 genomic DNA using primers S1 and S2. The SsXKS amplicon was ligated to the *Bam*HI/*Eco*RI site of p416Tef1-Ura3 (Runguphan and Keasling 2014) to yield pRS416Tef1-SsXKS.

*Plasmid pRS416Tef1*-*HXT7_F79S*: HXT7_F79S (accession number: NM_001180650) was amplified from *S. cerevisiae* BY4742 genomic DNA as two fragments using primers S3 and S4, and S5 and S6. S4 and S5 primers contain the point mutation required for the amino acid substitution. The two fragments were placed behind the P_TEF1_ promoter in p416Tef1-Ura3 to form pRS416Tef1-HXT7_F79S using homologous recombination in yeast.

*Plasmid pRS416Tef1*-*CtXYL1*: CtXYL1 (accession number: MF045434) was amplified from *C. tropicalis* BCC59435 genomic DNA using primers S7 and S8. The CtXYL1 amplicon was ligated to the *Bam*HI/*Eco*RI site of p416Tef1-Ura3 to yield pRS416Tef1-CtXYL1.

*Plasmid pRS416Tef1*-*CsXYL1*: CsXYL1 (accession number: MN200231) was synthesized by Genscript and was ligated into the *Bam*HI/*Eco*RI site of p416Tef1-Ura3 to yield pRS416Tef1-CsXYL1.

*Plasmid pRS416Tef1*-*SsXYL1*: SsXYL1 (accession number: HM769331) was amplified from *S. stipitis* BCC47637 genomic DNA using primers S9 and S10. The SsXYL1 amplicon was ligated to the *Bam*HI/*Sma*I site of p416Tef1-Ura3 to yield pRS416Tef1-SsXYL1.

*Plasmid pRS416Tef1*-*SpXYL1.1*: SpXYL1.1 (accession number: MN200233) was synthesized by Genscript and was ligated into the *Bam*HI/*Eco*RI site of p416Tef1-Ura3 to yield pRS416Tef1-SpXYL1.1.

*Plasmid pRS416Tef1*-*SpXYL1.2*: SpXYL1.2 (accession number: MN200234) was synthesized by Genscript and was ligated into the *Bam*HI/*Eco*RI site of p416Tef1-Ura3 to yield pRS416Tef1-SpXYL1.2.

*Plasmid pRS416Tef1*-*CtXYL2*: CtXYL2 (accession number: JN631039) was amplified from *C. tropicalis* BCC59435 genomic DNA using primers S11 and S12. The CtXYL2 amplicon was ligated to the *Sac*II/*Eco*RI site of p416Tef1-Ura3 to yield pRS416Tef1-CtXYL2.

*Plasmid pRS416Tef1*-*SsXYL2*: SsXYL2 (accession number: HM769332) was amplified from *S. stipitis* BCC47637 genomic DNA using primers S13 and S14. The SsXYL2 amplicon was ligated to the *Sac*II/*Eco*RI site of p416Tef1-Ura3 to yield pRS416Tef1-SsXYL2.

*Plasmid pRS416Tef1*-*SpXYL2*: SpXYL2 (accession number: MN200232) was synthesized by Genscript and was ligated into the *Sac*II/*Eco*RI site of p416Tef1-Ura3 to yield pRS416Tef1-SpXYL2.

*Plasmid pUG72*-*TDH3*-*LlkivDmit*-*2A*-*ScADH7mit*: P_TDH3_ promoter (accession number: NM_00118132) and ADH7 (accession number: NM_001178812) were amplified from *S. cerevisie* BY4742 genomic DNA using primers S15 and S16, and S17 and S18, respectively. LlkivD (codon-optimized for *S. cerevisiae* expression; accession number: MN200235) was synthesized by Genscript and provided in pUC57 vector. The gene was amplified from pUC57-LlkivD using primers S19 and S20. The three amplicons (P_TDH3_, LlkivD, ADH7) were joined together with pUG72 to form pUG72-TDH3-LlkivD-2A-ScADH7. To add the mitochondria signal peptide sequence to LlkivD and ADH7, P_TDH3_, LlkivD, ADH7 were amplified from pUG72-TDH3-LlkivD-2A-ScADH7 using primer S15 and S21, S22 and S23, and S24 and S18, respectively. The three amplicons (P_TDH3_, LlkivDmit, ADH7mit) were joined together with pUG72 to form pUG72-TDH3-LlkivDmit-2A-ScADH7mit.

*Plasmid pRS416Tef1*-*ScIlv2*-*2A*-*ScIlv5*-*2A*-*ScIlv3*: Ilv2 (accession number: NM_001182608), Ilv5 (accession number: NM_001182244) and Ilv3 (accession number: NM_001181674) were amplified from *S. cerevisiae* BY4742 genomic DNA using primers S25 and S26, S27 and S28, and S29 and S30, respectively. The three amplicons were placed behind the P_TEF1_ promoter in p416Tef1-Ura3 to form pRS416Tef1-ScIlv2-2A-ScIlv5-2A-ScIlv3 using homologous recombination in yeast.

*Plasmid pRSII416*-*TDH3*-*LlkivDmit*-*2A*-*ScADH7mit*: The TDH3-LlkivDmit-2A-ScADH7mit fragment was amplified from pUG72-TDH3-LlkivDmit-2A-ScADH7mit using primers S31 and S32. The amplicon was ligated into the *Apa*I/*Sma*I site of pRSII416 to yield pRSII416-TDH3-LlkivDmit-2A-ScADH7mit.

*Plasmids pRSII426Tef1*-*ScHXT7_F79S, pRSII426Tef1*-*SsXYL1, pRSII426Tef1*-*SsXYL2 and pRSII426Tef1*-*ScIlv2*-*2A*-*ScIlv5*-*2A*-*ScIlv3*-*Ura3*: The P_TEF1_-gene-T_CYC1_ cassette for each of the gene was amplified from its corresponding low-copy plasmid using primers S33 and S34. Each amplicon was individually ligated to the *Not*I/*Sac*I site of pRSII426 to yield the high-copy plasmid.

*Plasmid pRSII426Tef1*-*SsXKS*: The P_TEF1_-SsXKS-T_CYC1_ cassette was amplified from pRS416Tef1-SsXKS-Ura3 using primers S35 and S36. The amplicon was ligated into the *Pst*I/*Not*I site of pRSII426 to yield pRSII426Tef1-SsXKS.

*Plasmid pRSII426*-*TDH3*-*LlkivDmit*-*2A*-*ScADH7mit*: The P_TDH3_-LlkivDmit-2A-ScADH7mit-T_CYC1_ cassette was amplified from pRSII416-TDH3-LlkivDmit-2A-ScADH7mit using primers S37 and S34. The amplicon was ligated into the *Not*I/*Sac*I site of pRSII426 to yield pRSII426-TDH3-LlkivDmit-2A-ScADH7mit.

### Screening of XR–XDH variants in *S. cerevisiae*

Engineered strains harboring different combination of XR–XDH variants were pre-cultured in 5-mL aliquots in SD selective yeast medium (6.7 g/L of Yeast Nitrogen Base (Difco), 20 g/L glucose, and a mixture of appropriate nucleotide bases and amino acids (CSM) overnight and used to inoculate 10 mL SX selective yeast medium (6.7 g/L of Yeast Nitrogen Base (Difco), 20 g/L xylose, and a mixture of appropriate nucleotide bases and amino acids (CSM) to achieve an initial optical density of 0.05 at 600 nm (OD_600_). The cultures were grown at 30 °C and 250 rpm in an orbital shaking incubator. Samples were taken at 24, 48 and 72 h time points for OD_600_ measurement.

### Quantification of isobutanol production in engineered strains

Engineered yeast strains were grown overnight in 5 mL of SD selective yeast medium in a 50 mL Falcon tube. Then, the cultures were inoculated into 50 mL of SX selective yeast medium in 250-mL flask cultures to achieve an initial OD_600_ of 0.05. The cultures were grown at 30 °C and 250 rpm in an orbital shaking incubator. Samples were taken at 72 and 144 h to determine OD_600_, biomass, extracellular metabolites and production of higher alcohols. The amount of isobutanol and other extracellular metabolites were determined using high-performance liquid chromatography (HPLC) as previously reported with small modifications (Avalos et al. 2013; Siripong et al. 2018). Briefly, 1 mL of culture was centrifuged at 18,000*g* for 5 min and the supernatant was filtered through 0.2 μm nylon syringe filter (Filtrex). The purified sample was then applied to an Agilent 1100 series HPLC equipped with an Aminex HPX-87H ion exchange column (Biorad). The LC program was performed using 5 mM H_2_SO_4_ as the solvent at a flow rate of 0.68 mL/min for 30 min. The column was maintained at 60 °C. All metabolites were detected with Agilent 1200 series DAD and RID detectors.

## Results

### Screening of optimal xylose reductase–xylose dehydrogenase pathway genes

To engineer *S. cerevisiae* with the ability to grow on xylose, we first integrated the xylose-specific variant of the sugar transporter *HXT7* (*HXT7*_F79S_) and a copy of the *S. stipitis* xylulokinase gene (*XYL3*) into BY4742, a laboratory strain of *S. cerevisiae*. While a gene encoding xylulokinase is present in *S. cerevisiae* (i.e. *XKS1*), its expression level is insufficient for efficient conversion of xylulose to xylulose-5-phosphate (Johansson et al. 2001). The resulting strain, PWY0013, while still unable to grow in selective medium containing xylose as the sole carbon source, provided the necessary background for XR–XDH screening (Fig. 1, Table 2). We screened different XR and XDH candidate enzymes that have demonstrated high activity in *S. cerevisiae* (Kim et al. 2013; Guo et al. 2016; Li et al. 2016). For XR, these are *Ct*XYL1 from *Candida tropicalis*, *Cs*XR from *Candida shehatae*, *Ss*XYL1 from *S. stipitis*, *Sp*XYL1.1 and *Sp*XYL1.2 from *Spathaspora passalidarum*. The first four enzymes prefer NADPH as the redox cofactor while the last enzyme prefers NADH. For XDH, these are *Ct*XDH from *C. tropicalis*, *Ss*XYL2 from *S. stipitis*, and *Sp*XYL2 from *S. passalidarum*. To ensure robust and stable expression of these genes, we integrated the individual XR and XDH constructs into the *ARS308* and *ARS720* sites, respectively. These chromosomal integration sites have previously been shown to lead to high expression level of the inserted gene (Reider Apel et al. 2017).

**Table 2:** Overview of different microbial strains engineered to produce isobutanol (specific growth rates and isobutanol titer) including those that were generated in this study

Strain name	Genotype	Description	Specific growth rate in glucose (h^−1^)	Specific growth rate in xylose (h^−1^)	Isobutanol titer in glucose (m/L)^a^	Isobutanol titer in xylose (m/L)^a^	Isobutanol productivity in xylose (mg/gCDW^−1^h^−1^)	References
Isoy16	*ilv2Δ0; ilv5Δ0; ilv3Δ0* p425-synthILV235, pRS42HH7-Aro10-Ash2, pRS42NH7-Tal1-Xks1, pRS24H7-synthXI	CEN.PK with deletions in *Ilv2, Ilv5* and *Ilv3* overexpressing XylA from *C. phytofermentans*, *XKS1* and *Aro10* from *S. cerevisiae* and cytosol-targeted *Ilv2*, *Ilv5* and *Ilv3* from *S. cerevisiae*	Not reported	Not reported	Not reported	1.36 ± 0.11^b^	Not reported	Brat and Boles (2013)
BY4742	*Mat α; his3Δ1; leu2Δ0; lys2Δ0; ura3Δ0*	None	0.1395 ± 0.0003	N.D.	4.0 ± 0.5	N.D.	N.D.	This study
PWY0013	BY4742 *ARS208::P*_*TEF1*_-*SsXKS; YMRWΔ15::P*_*TEF1*_-*HXTF79S*	BY4742 overexpressing *XKS* from *Scheffersomyces stipitis*, *HXT7*_F79S from *Saccharomyces cerevisiae*	0.1329 ± 0.0001	N.D.	5.5 ± 0.7	N.D.	N.D.	This study
PWY1113	PWY0013 *ARS308::P*_*TEF1*_-*CtXYL1; ARS720::P*_*TEF1*_-*CtXYL2*	PWY0013 overexpressing *XYL1* and *XYL2* from *Candida tropicalis*	0.1435 ± 0.0004	0.0313 ± 0.0011	2.8 ± 0.1	3.8 ± 0.4	0.047 ± 0.005	This study
PWY2313	PWY0013 *ARS308::P*_*TEF1*_-*SsXYL1; ARS720::P*_*TEF1*_-*SsXYL2*	PWY0013 overexpressing *XYL1* and *XYL2* from *S. stipitis*	0.1461 ± 0.0003	0.0423 ± 0.0004	5.0 ± 0.4	4.3 ± 0.7	0.052 ± 0.008	This study
PWY1123	PWY1113 *GRE3Δ*	PWY1113 with *GRE3* deleted	0.1472 ± 0.0003	0.0295 ± 0.0012	13.9 ± 0.9	5.7 ± 0.6	0.076 ± 0.008	This study
PWY2323	PWY2313 *GRE3Δ*	PWY2313 with *GRE3* deleted	0.1436 ± 0.0002	0.0426 ± 0.0001	23.5 ± 0.7	4.4 ± 0.4	0.053 ± 0.005	This study
PWY1143	PWY1123 *ARS1309::P*_*TEF1*_-*ScIlv2*-*T2A*-*ScIlv5*-*T2A*-*ScIlv3; YORW Δ22::P*_*TDH3*_ –*LlkivDmt*-*T2A*-*ScADH7mt*	PWY1123 overexpressing *Ilv2, Ilv5, Ilv3, ADH7* from *S. cerevisiae* and *kivD* from *L. lactis*	0.1463 ± 0.0003	0.0368 ± 0.0015	30.4 ± 1.0	9.4 ± 0.3	0.114 ± 0.006	This study
PWY2343	PWY2323 *ARS1309::P*_*TEF1*_-*ScIlv2*-*T2A*-*ScIlv5*-*T2A*-ScIlv3; *YORW Δ22::P*_*TDH3*_ –*LlkivDmt*-*T2A*-*ScADH7mt*	PWY2323 overexpressing *Ilv2, Ilv5, Ilv3, ADH7* from *S. cerevisiae* and *kivD* from *L. lactis*	0.1476 ± 0.0004	0.0431 ± 0.0004	43.3 ± 1.0	16.9 ± 1.9	0.226 ± 0.014	This study
PWY1153	PWY1143 *PHO13Δ*	PWY1143 with *PHO13* deleted	0.1408 ± 0.0003	0.0513 ± 0.0002	33.9 ± 1.2	11.8 ± 0.8	0.134 ± 0.010	This study
PWY2353	PWY2343 *PHO13Δ*	PWY2343 with *PHO13* deleted	0.1469 ± 0.0003	0.0532 ± 0.0004	43.0 ± 0.9	19.7 ± 2.4	0.172 ± 0.016	This study

N.D. represents not detected due to strain’s inability to grow

^a^Isobutanol titers after 2 days of cultivation in yeast selective medium

^b^Isobutanol titers after 6 days of cultivation in yeast selective medium

**Fig. 2:**
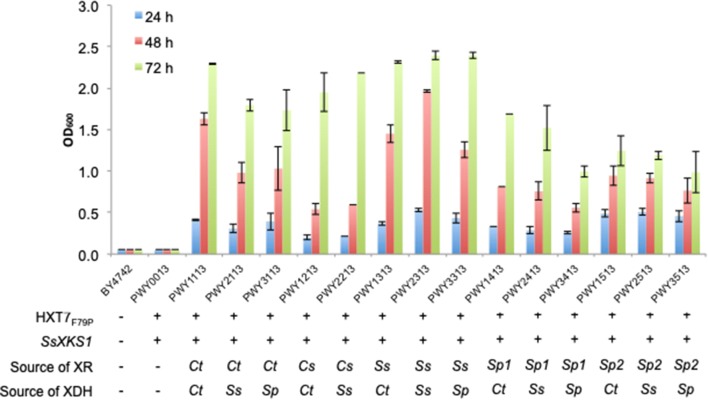
Growth profile of engineered *S. cerevisiae* harboring different XR–XDH variants in *S. cerevisiae.* Engineered strains were pre-cultured in 5-mL aliquots in yeast selective medium (2% glucose) overnight and used to inoculate 10 mL yeast selective medium (2% xylose) to achieve an initial optical density of 0.05 at 600 nm (OD_600_). The cultures were grown at 30 °C and 250 rpm in an orbital shaking incubator. Samples were taken at 24, 48 and 72 h time points for OD_600_ measurement. Values are the mean of three biological replicates ± standard deviation (n = 3). *Ct*, *Candida tropicalis*; *Cs*, *Candida shehatae*; *Ss*, *Scheffersomyces stipitis*; *Sp*, *Spathaspora passalidarum*; *Sp1*, *Spathaspora passalidarum XYL1.1*; *Sp2*, *Spathaspora passalidarum XYL1.2*

The engineered strains exhibited superior cell growth when compared to strains BY4742 (Baker Brachmann et al. 1998) and PWY0013 (Fig. 2, Table 2). Overall, the choice of XR appeared to affect cell growth more so than the choice of XDH. In particular, strains expressing XR from *S. stipitis* performed the best, reaching the final OD_600_ (after 72 h) of 2.32–2.40, while strains harboring either XR variants from *S. passalidarum* performed the worst, reaching the final OD_600_ of 0.99–1.69. Interestingly, strains harboring XR from *C. shehatae* grew slowly within the first 48 h, reaching an OD_600_ of ~ 0.5, but then grew rapidly in the final 24 h to reach an OD_600_ of 2.0–2.2. Despite several attempts, we were unable to obtain a viable strain overexpressing XR from *C. shehatae* and XDH from *S. passalidarum*. Strains PWY2313, which harbors XR and XDH from *S. stipitis*, and PWY1113, which harbors XR and XDH from *C. tropicalis*, achieved the highest OD_600_ after 48 h at 1.96 and 1.63, respectively, and were used in subsequent engineering steps.

### Integration of isobutanol pathway genes for production of isobutanol from xylose

To engineer isobutanol production in the yeast *S. cerevisiae*, we integrated the non-fermentative isobutanol pathway that combines the l-valine biosynthetic and ketoacid degradation pathways (Fig. 1). In deciding the localization of the isobutanol pathway enzymes, we contend that targeting the five enzymes to the mitochondria is a more appropriate strategy. Growth on xylose and other non-fermentable sugars has been shown to dis-regulate glucose-dependent repression of mitochondria development and induce its metabolism (DeRisi et al. 1997; Egner et al. 2002). For the choice of the keto-acid degradation pathway enzymes, we chose the keto-acid decarboxylase LlkivD from *L. lactis* and the endogenous alcohol dehydrogenase ADH7 based on a previous report by Avalos and co-workers that demonstrated robust activities of the two enzymes when they were both targeted to the mitochondria (Avalos et al. 2013). To incorpotate the isobutanol pathway into PWY1113 and PWY2313, we linked genes from each pathway together with the self-cleaving 2A peptide sequence from the foot-and-mouth disease virus (FMDV) (Ryan and Drew 1994) and separately placed them behind the strong constitutive promoters P_TEF1_ and P_TDH3_. This ensures stoichiometric production of all the proteins whose genes are linked together by the 2A peptide sequence. The mitochondrial signal peptide sequence was added to *LlkivD* and *ADH7* to target the enzymes to the mitochondria. Finally, to increase xylose utilization, we deleted the endogenous aldose reductase gene *GRE3* as well as the alkaline phosphatase gene *PHO13*. These deletions have been used to improve xylose utilization in *S. cerevisiae* expressing either the XR–XDH or XI pathway (Karhumaa et al. 2007).

**Fig. 3:**
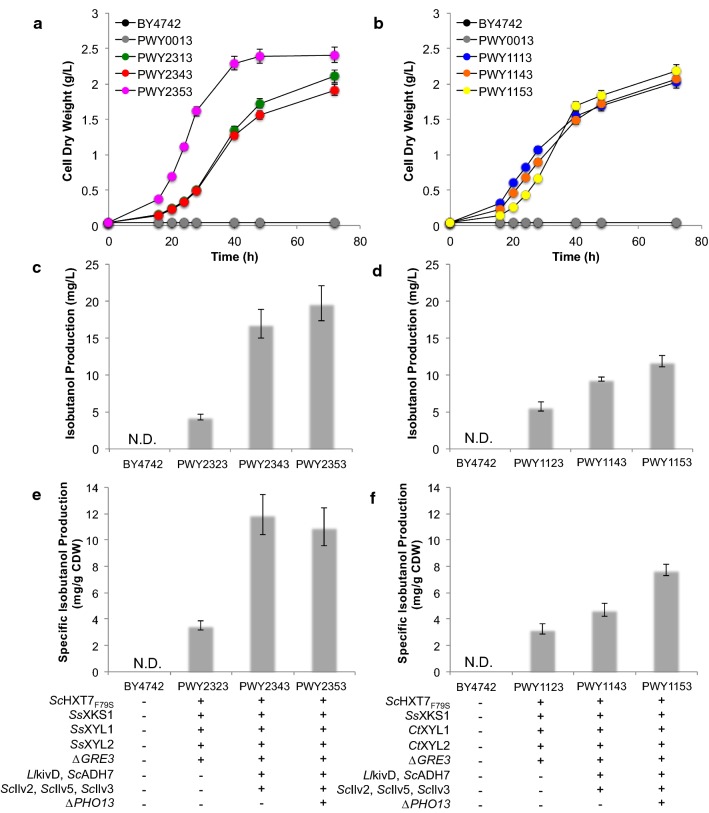
Growth profile and isobutanol production of engineered strains in selective medium containing 2% xylose. Growth profiles of engineered strains expressing XR–XDH from *S. stipitis* (**a**) and *C. tropicalis* (**b**). Total (**c**) and specific (**e**) isobutanol production after 2 days of engineered strains expressing XR–XDH from *S. stipitis*. Total (**d**) and specific (**f**) isobutanol production after 2 days of engineered strains expressing XR–XDH from *C. tropicalis*. Engineered strains were pre-cultured in 5-mL aliquots in SD selective medium overnight and used to inoculate 10 mL fresh SX (2% xylose) to achieve an initial optical density of 0.05 at 600 nm (OD_600_). The cultures were grown at 30 °C and 250 rpm in an orbital shaking incubator. Samples were taken after 2 days and the supernatants were analyzed on HPLC to quantify the isobutanol content. Values are the mean of three biological replicates ± standard deviation (n = 3). N.D. represents not detected due to strain’s inability to grow. *Ll, Lactococcus lactis*; *Sc*, *Saccharomyces cerevisiae*; *Ss*, *Scheffersomyces stipitis*; *Ct*, *Candida tropicalis*

Gratifyingly, we observed isobutanol production in our engineered strains (Fig. 3, Table 2). Overall, strains overexpressing XR and XDH from *S. stipitis* performed better, resulting in greater cell growth and higher isobutanol levels. In particular, strain PWY2343, which expresses XR and XDH from *S. stipitis* as well as the complete isobutanol pathway, produced 16.9 ± 1.9 mg/L of isobutanol (specific isobutanol production of 11.9 ± 1.5 mg/g CDW; specific growth in xylose of 0.0431 ± 0.0004 h^−1^) after 2 days of cultivation. The deletion of *PHO13* in strain PWY2343 to create strain PWY2353 further improved isobutanol titer to 19.7 ± 2.4 mg/L (specific isobutanol production of 11.0 ± 1.4 mg/g CDW; specific growth in xylose of 0.0532 ± 0.0004 h^−1^). In comparison, the most effective strain expressing XR and XDH from *C. tropicalis* (strain PWY1353) produced isobutanol at a titer of only 11.8 ± 0.8 mg/L (specific isobutanol production of 7.7 ± 0.4 mg/g CDW; specific growth in xylose of 0.0513 ± 0.0002 h^−1^).

### Improving isobutanol titer by fine-tuning the expression levels of the biosynthetic pathway genes

To further improve the isobutanol production titer in PWY2353, we turned our attention to fine-tuning the expression levels of the biosynthetic pathway genes. Specifically, we studied the effect of additional copies of the XR–XDH pathway genes, the isobutanol pathway genes, as well as the transporter and xylulokinase genes. We used the CEN6/ARS4 and 2 µm plasmid-expression for low and high-copy expression, respectively (Chee and Haase 2012). The yeast CEN6/ARS4 origin of replication should result in transformants with a low copy plasmid number (1 copy per cell). In contrast, the yeast 2 μ origin of replication allows autonomous replication of the plasmids and is expected to result in transformants with a high plasmid copy number (10–40 copies per cell). We also extended fermentation time to 6 days to increase isobutanol titer as well as to allow a more direct comparison to the previous report by Brat and Boles (2013).

**Fig. 4:**
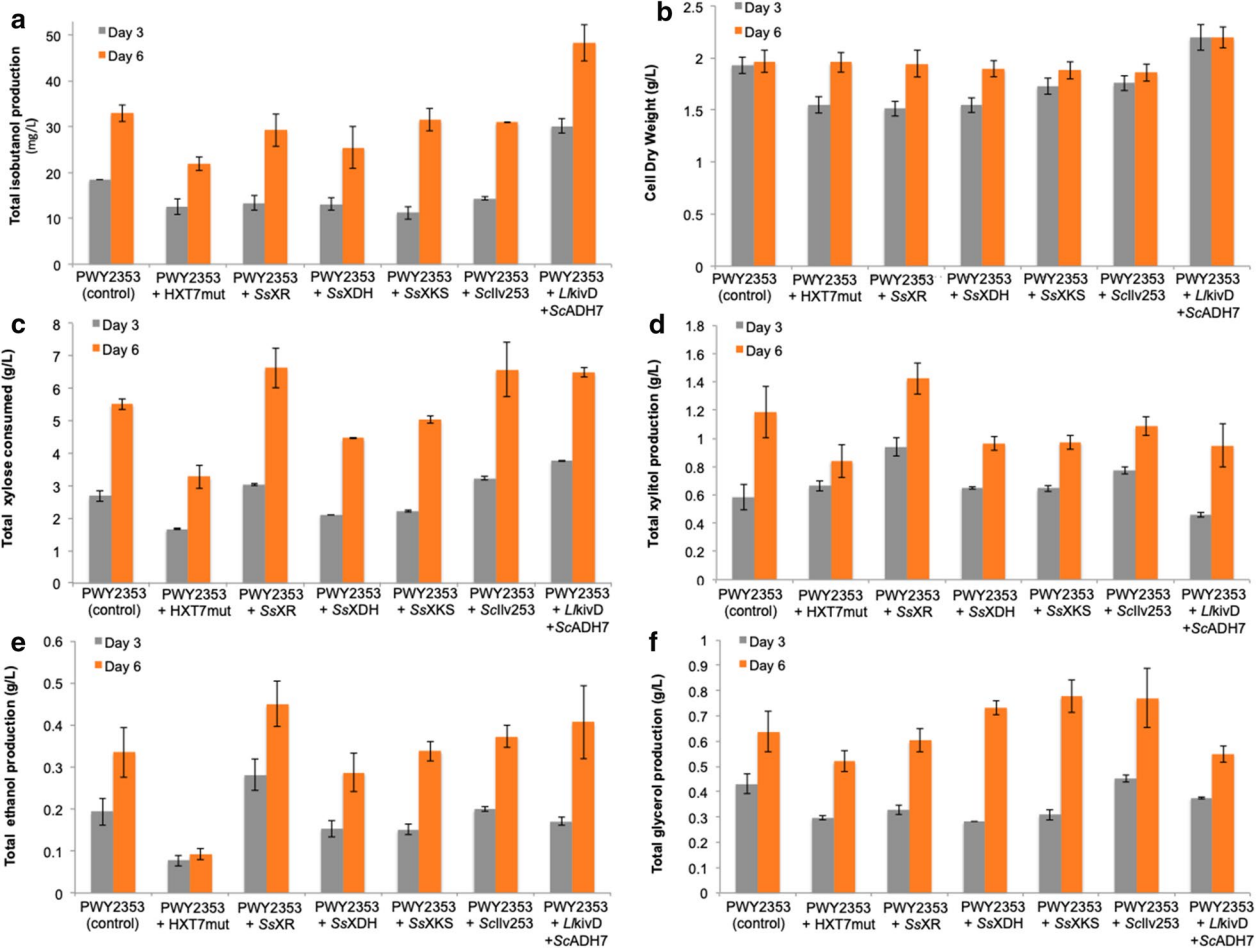
Characterization of engineered strains with high-copy (2 µm) plasmid-based overexpression of individual pathway genes. Total isobutanol production (**a**), cell dry weight (**b**), xylose consumption (**c**), xylitol production (**d**), ethanol production (**e**), and glycerol production (**f**). Individual gene construct was placed in the pRSII426 plasmid and transform into PWY2353. The resulting strains were cultured in selective medium containing 2% xylose as the sole carbon source. Values are the mean of three biological replicates ± standard deviation (n = 3) after 3 and 6 days

We obtained mixed success with high-copy plasmid-based overexpression (Fig. 4). Notably, high-copy plasmid-based expression of *Ll*kivD and *Sc*ADH7 led to the highest total isobutanol production at 47.7 ± 5.0 mg/L, a 45% increase from the control strain (PWY2353 harboring an empty high-copy vector) at 32.9 ± 1.7 mg/L after 6 days of cultivation. This improved strain (PWY2343 harboring pRSII426-*Ll*kivD-*Sc*ADH7) consumed more xylose and produced lower levels of the side products glycerol and xylitol. In contrast, additional copies of either HXT7_F79S_ or SsXDH resulted in less xylose consumption as well as decreases in total isobutanol production (21.9 ± 1.5 mg/L and 25.5 ± 4.6 mg/L, respectively).

**Fig. 5:**
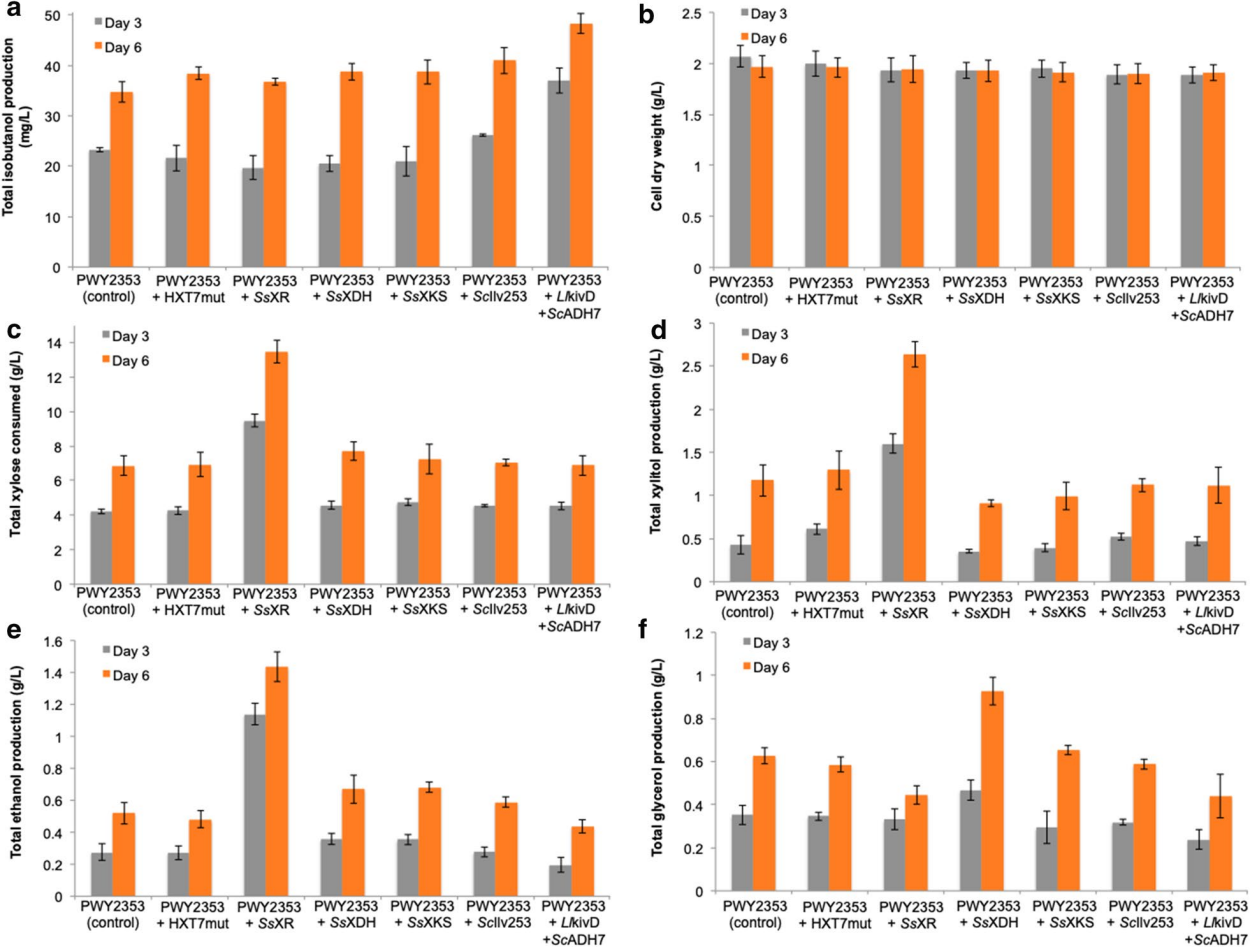
Characterization of engineered strains with low-copy plasmid-based overexpression of individual pathway genes. Total isobutanol production (**a**), cell dry weight (**b**), xylose consumption (**c**), xylitol production (**d**), ethanol production (**e**), and glycerol production (**f**). Individual gene construct was placed in the pRSII416 plasmid and transform into PWY2353. All strains were cultured in selective medium containing 2% xylose as the sole carbon source. Values are the mean of three biological replicates ± standard deviation (n = 3) after 3 and 6 days

We observed similar results with low-copy plasmid-based expression (Fig. 5). Specifically, overexpression of *Ll*kivD and *Sc*ADH7 improved isobutanol titer to 48.4 ± 2.0 mg/L (specific isobutanol production of 25.3 ± 2.8 mg/g CDW; yield of 7.0 mg/g consumed d-xylose), which is 40% higher than in the control strain (PWY2343 harboring an empty vector) and approximately 36-folds higher than that from the previous report by Brat and Boles (total isobutanol production of 1.36 ± 0.11 mg/L after 146 h of cultivation). The increase in isobutanol of this strain (PWY2343 harboring pRSII416-*Ll*kivD-*Sc*ADH7) was accompanied by a decrease in the formation of the side products ethanol and glycerol (Fig. 5), while biomass, xylose consumption and xylitol formation remained unchanged. Accumulatively, our results from both high-copy and low-copy plasmid-based overexpression indicate that overexpression of the terminal enzymes (i.e. those leading directly to the final product isobutanol) had the largest effect on isobutanol titers.

Another engineered strain of note is PWY2353 overexpressing *Ss*XR (PWY2353 harboring pRSII416-*Ss*XR), which had the highest xylose consumption at 13.0 ± 0.5 g/L xylose, roughly twice the amount consumed in the control strain (at 6.8 ± 0.6 g/L). However, this increase in xylose consumption did not improve isobutanol production, but rather led to increases in the formation of the pathway intermediate xylitol (2.6 ± 0.2 g/L up from 1.2 ± 0.2 g/L) and the side product ethanol (1.4 ± 0.1 g/L up from 0.5 ± 0.1 g/L). XR catalyzes the reduction of xylose to xylitol and accumulation of xylitol indicates that the next enzyme in the pathway, xylitol dehydrogenase (XDH) may be limited. Accumulation of ethanol indicates that the reduction of xylose to xylitol by XR potentially caused redox cofactor imbalances. Therefore, further strain improvement may be achieved by overexpression of an engineered XDH that has a cofactor preference for NADP^+^ (Ehrensberger et al. 2006; Watanabe et al. 2007). Alternatively, down-regulation of the ethanol pathway by deleting the pyruvate decarboxylase gene PDC1 and the alcohol dehydrogenase genes ADH1-5 may further improve the isobutanol titer (Baek et al. 2017).

## Discussion

Recent work on the design and construction of robust cell factories for efficient conversion of xylose into biofuels can be grouped into four complementary strategies: (1) introduction of a xylose assimilation pathway that is stoichiometrically advantageous with regards to the biofuel of interest; (2) introduction of a xylose-specific transporter; (3) introduction of specific gene knockouts to improve xylose utilization; and (4) incorporation of the desired biofuel pathway (Kwak and Jin 2017; Li et al. 2019). We designed our isobutanol producing strain by combining these strategies.

For the first strategy, we used the conventional xylose reductase–xylose dehydrogenase pathway as it has a higher pyruvate yield based on stoichiometry when compared with the xylulose-1-phosphate (X-1-P) pathway (1.67 vs. 1 mol/mol xylose) (Cam et al. 2016). The synthetic X-1-P pathway, which converts xylose into xylulose-1-phosphate, before an aldolytic cleavage produces glycolaldehyde and dihydroxyacetone phosphate (DHAP), is more advantageous for the production of C2 chemicals such as ethylene glycol (Chomvong et al. 2016). The xylose isomerase (XI) pathway, while having a similar pyruvate yield as the XR–XDH pathway, suffers from low catalytic efficiency of the isomerase enzyme as well as the difficulty of functionally expressing bacterial XI enzymes in *S. cerevisiae* (Sarthy et al. 1987; Amore et al. 1989; Moes et al. 1996). To determine the optimal XR–XDH combination, we screened different XR and XDH variants from several xylose-utilizing yeasts.

For the second strategy, we introduced a re-engineered hexose transporter HXT7 into *S. cerevisiae* (Reider Apel et al. 2016). Apel and coworkers discovered a F79S point mutation in HXT7 that enables the hexose transporter to transport xylose into the cell with high efficiency. For the third strategy, we disrupted the aldose reductase gene *GRE3* and the alkaline phosphatase *PHO13* as previous reports indicated that deletion of these genes improved xylose utilization (Parreiras et al. 2014; Xu et al. 2016). Finally, for the last strategy, we overexpressed mitochondria-targeted isobutanol pathway enzymes. Applying all of these strategies resulted in strain PWY2353, which is able to produce isobutanol at a titer of 19.7 ± 2.4 mg/L in 48 h (specific isobutanol production of 11.0 ± 1.4 mg/g CDW; specific growth in xylose of 0.0532 ± 0.0004 h^−1^).

We further improved strain PWY2353 by fine-tuning the expression levels of the individual biosynthetic pathway genes. A large body of work in metabolic engineering indicates that the expression levels of metabolic pathway genes greatly impact the production levels of the compound of interest (Lee et al. 2012). Too low expression levels lead to bottlenecks while too high expression levels can deplete the cellular resources that could otherwise be used to produce the compound of interest. Fine-tuning of the pathway enzyme expression levels was achieved by adding additional gene copies via the use of CEN6/ARS4 (low-copy) and 2 µm (high-copy) plasmid expression systems. In both systems, we observed the biggest improvements in isobutanol production when the final two enzymes (*Ll*kivD and *Sc*ADH7) were overexpressed. Notably, this “pulling” on the pathway by expressing the terminal enzyme is a common and effective strategy to pull flux towards the desired product as part of the “push–pull-block” strategy (Tai and Stephanopoulos 2013). Our best producer, strain PWY2353 with additional low-copy plasmid-based expression of *Ll*kivD and *Sc*ADH7, was able to produce isobutanol at a titer of 48.4 ± 2.0 mg/L after 6 days of cultivation (specific isobutanol production of 25.3 ± 2.8 mg/g CDW; yield of 7.0 mg/g consumed d-xylose). To our knowledge, these are the highest isobutanol titer and yield from xylose reported in any microorganism, natural or engineered. While these values are lower when compared to isobutanol production systems based on glucose, given that xylose comprises a large portion of biomass, our work provides a foundational step towards more sustainable and efficient production of advanced biofuels.

## Supplementary information

**Additional file 1.** Information about primers and plasmids used in this study.

## Data Availability

All datasets on which the conclusions of the manuscript rely are presented in the main paper and additional information.
