# Supplementary material for: Systematic improvement of isobutanol production from d-xylose in engineered Saccharomyces cerevisiae
Source: AMB Express. 2019 Oct 10;9:160. doi: 10.1186/s13568-019-0885-3 (PMC6787123; doi:10.1186/s13568-019-0885-3)
Supplement: Supplementary file 1 — Additional file 1. Information about primers and plasmids used in this study. [file 13568_2019_885_MOESM1_ESM.docx]

**Additional Information for Systematic Improvement of Isobutanol Production from D-xylose in Engineered *Saccharomyces cerevisiae***

Peerada Promdonkoy^1^, Wiparat Siripong^1^, Joe James Downes^2,3^, Sutipa Tanapongpipat^1^, and Weerawat Runguphan^1^

^1^National Center for Genetic Engineering and Biotechnology, 113 Thailand Science Park, Paholyothin Road, Klong 1, Klong Luang, Pathumthani 12120, Thailand

^2^University of Kent, Canterbury, Kent, UK

^3^ Present address: Syngenta, Jealott’s Hill International Research Station, Bracknell, Berkshire RG42 6EY, UK

Corresponding Author

Tel: +66 02-564-6700, Fax: +66 02-564-6701

Address: National Center for Genetic Engineering and Biotechnology, 113 Thailand Science Park, Paholyothin Road, Klong 1, Klong Luang, Pathumthani 12120, Thailand

E-mail: weerawat.run@biotec.or.th

**1. Primers used in this study**

**1.1 Primers for gene deletions**

**Primer for *GRE3* deletion**

Forward

5’ TAATATAAATCGTAAAGGAAAATTGGAAATTTTTTAAAGATGCATAGGCCACTAGTGGAT 3’

Reverse

5’ TGTTCATATCGTCGTTGAGTATGGATTTTACTGGCTGGATCACAGCTGAAGCTTCGTACG 3’

**Primer for *PHO13* deletion**

Forward

5’ TTATAGCTTGCCCTGACAAAGAATATACAACTCGGGAAAATGCATAGGCCACTAGTGGAT 3’

Reverse

5’ TCAAAAAGTAATTCTACCCCTAGATTTTGCATTGCTCCTCTACAGCTGAAGCTTCGTACG 3’

**1.2 Primers for plasmid construction**

Bold nucleotides designate the Kozak sequence; underlined nucleotides are restriction sites

**pRS416Tef1-SsXKS1**

S1. Forward

5’ ATAGGATCC**AAAACA**ATGACCACTACCCCATTTG 3’

S2. Reverse

5’ ATAGAATTCTTAGTGTTTCAATTCACTTTCC 3’

**pRS416Tef1-HXT7_F79S**

S3. Forward

5’ GCAATCTAATCTAAGTTTTCTAGAACTAGTGGATCC**AAAACA**ATGTCACAAGACGCTGCT 3’

S4. Reverse

5’ CCAGTATCCCAACCGGAAACGAAACCACCAAAGG 3’

S5 Forward

5’ TTGGTGGTTTCGTTTCCGGTTGGGATACTGGTAC 3’

S6 Reverse 2

5’ GAGGTCGACGGTATCGATAAGCTTGATATCGAATTCTTATTTGGTGCTGAACATTCT 3’

**pRS416Tef1-CtXYL1**

S7 Forward

5’ ATAGGATCC**AAAACA**ATGTTTAAATTTTTCACTTCTC

S8 Reverse

ATAGAATTCTTAAACAAAGATTGGAATGTTG

**pRS416Tef1-SsXYL1**

S9 Forward

5’ ATAGGA TCC**AAAACA**ATGCCTTCTATTAAGTTGAAC 3’

S10 Reverse

5’ ATACCCGGGTTAGACGAAGATAGGAATCTT 3’

**pRS416Tef1-CtXYL2**

S11 Forward

5’ ATATATCCGCGG**AAAACA**ATGACTGCAAACCCATCA 3’

S12 Reverse

5’ ATATATGAATTCCTATTCTGGACCATCAATTAAAC 3’

**pRS416Tef1-SsXYL2**

S13 Forward

5’ ATATATCCGCGG**AAAACA**ATGACTGCTAACCCTTCC 3’

S14 Reverse

5’ ATATATGAATTCTTACTCAGGGCCGTCAAT 3’

**pUG72-TDH3-LlkivDmit-2A-ScADH7mit**

S15 Forward

5’ ATACGACTCACTATAGGGAGACCGGCAGATCCGCGGGAGTTTATCATT 3’

S16 Reverse

5’ AGTGTACATTGTTTTCCGCGGTTTGTTTGTTTA 3’

S17 Forward

5’ GGTTCTTTGTTGACTTGTGGAGATGTTGAGGAGAATCCAGGACCAATGCTTTACCCAGAAAAATTT 3’

S18 Reverse

5’ GACGGTATCGATAAGCTTGATATCGAATTCCTATTTATGGAATTTCTTATCATA 3’

S19 Forward

5’ ACAAACAAACCGCGGAAAACAATGTACACTGTCGGA 3’

S20 Reverse

5’ CTCAACATCTCCACAAGTCAACAAAGAACCCCTTCCTTCTGCTCTGGATTTGTTCTGTTCTGCGAA 3’

S21 Reverse

5’ AAGTTCTTGTGGCTGGCTTGAAAAATCTTATAGATTGACGTAGTGAAAGCATTGTTTTCCGCGGTTT 3’

S22 Forward

5’ ATTTTTCAAGCCAGCCACAAGAACTTTGTGTAGCTCTAGATATCTGCTTTACACTGTCGGAGATTAC 3’

S23 Reverse

5’ aAGTTCTAGTAGCAGGCTTAAAGAAtCTTATAGATTGACGTAGTGAAAGCATTGGTCCTGGATTCTC 3’

S24 Forward

5’ ATTCTTTAAGCCTGCTACTAGAACTtTGTGTAGCTCTAGATATCTGCTTCTTTACCCAGAAAAATTTCAG 3’

**pRS416Tef1-ScIlv2-2A-ScIlv5-2A-ScIlv3-Ura3**

S25 Forward

5’ CTTGCTCATTAGAAAGAAAGCATAGCAATCTAATCTAAGTTTTCTAGAACTAGTGGATCCAAAACAATGATCA GACAATCTACGCTA 3’

S26 Reverse

5’ TGGTCCTGGATTCTCCTCAACATCTCCACAAGTCAACAAAGAACCCCTTCCTTCTGCTCTGTGCTTACCGCCTGTACG 3’

S27 Forward

5’ AGAGCAGAAGGAAGGGGTTCTTTGTTGACTTGTGGAGATGTTGAGGAGAATCCAGGACCATTGAGAACTCAAGCCGCC 3’

S28 Reverse

5’ AGGACCAGGGTTTTCTTCTACGTCACCGCATGTTAGTAGACTTCCTCTACCCTCAGCTCTTTGGTTTTCTGGTCTCAACTT 3’

S29 Forward

5’ AGAGCTGAGGGTAGAGGAAGTCTACTAACATGCGGTGACGTAGAAGAAAACCCTGGTCCTGGCTTGTTAACGAAAGTTGCT 3’

S30 Reverse

5’ GTGACATAACTAATTACATGACTCGAGGTCGACGGTATCGATAAGCTTGATATCGAATTCTCAAGCATCTAAAACACAACC 3’

**Plasmid pRSII416-TDH3-LlkivDmit-2A-ScADH7mit**

S31 Forward

5’ ATATGGGCCCGAGTTTATCATTATCAATACTGCC 3’

S32 Reverse

5’ ATATCCCGGGGGCCGCAAATTAAAGCCT 3’

**Plasmids pRSII426Tef1-ScHXT7_F79S, pRSII426Tef1-SsXYL1, pRSII426Tef1-SsXYL2 and pRSII426Tef1-ScIlv2-2A-ScIlv5-2A-ScIlv3-Ura3**

S33 Forward

5’ ATATATGCGGCCGCATAGCTTCAAAATGTTTCTACTC 3’

S34 Reverse

5’ ATATATGAGCTCGGCCGCAAATTAAAGCCT 3’

**pRSII426Tef1-SsXKS**

S35 Forward

5’ ATATATCTGCAGATAGCTTCAAAATGTTTCTACTC 3’

S36 Reverse

5’ ATATATGCGGCCGCGGCCGCAAATTAAAGCCT 3’

**Plasmid pRSII426-TDH3-LlkivDmit-2A-ScADH7mit**

S37 Forward

5’ ATATATGCGGCCGCGAGTTTATCATTATCAATACTGCC 3’

**1.3 Primers for chromosomal integration**

**Integration into *ARS208* site**

Forward

5’ AGGGACCATACACACGTCCGCTAAACAAAAGATCTTGGGCATCAGCTGAAGCTTCGTACG 3’

Reverse

5’ CCCTCTTTTCCACCCCGTGAAAAACAGAATATCATTAACCGGACGACTCACTATAGGGCG 3’

**Integration into *YMRWΔ15* site**

Forward

5’ GATGACTGTTTCTCAAACTTTATGTCATTTTCTTACACCGCACAGCTGAAGCTTCGTACG 3’

Reverse

5’ ACCGCGAAGATTTATAATGGTTTATCGGTTGCATTTTCCATGACGACTCACTATAGGGCG 3’

**Integration into *ARS308* site**

Forward

5’ GAAATTTCAACATTAACTTCGAATTTTTTTCTTTTTATCTAACAGCTGAAGCTTCGTACG 3’

Reverse

5’ TAGAAGTGGTAGCAATATGTAGCAAAGAAGACAAGTAATCCTACGACTCACTATAGGGCG 3’

**Integration into *ARS720* site**

Forward

5’ GTTACTGTTGATTGTTCGTTTATTTGTATAATTGAGTTTACACAGCTGAAGCTTCGTACG 3’

Reverse

5’ ATTTATAAGTTTGCTTTTTGTCACTCTCTTGGCCCTAATTACACGACTCACTATAGGGCG 3’

**Integration into *ARS1309* site**

Forward

5’ ATTCTAGTATCAAAGAAACTTACTATGACGCAGTTTAGGATCCAGCTGAAGCTTCGTACG 3’

Reverse

5’ ACACTGAATAAACAAGGGGCTTTACGATGGAGTAGTAGACCTACGACTCACTATAGGGCG 3’

**Integration into *YORWΔ22* site**

Forward

5’ CACCGGAGCTTGGATATGATAAACGAAATATTCTTGAATCGTCCGCGGGAGTTTATCATT 3’

Reverse

5’ CGTGATAAACGATCGCCATAACTAACAGGTATAAATGGCAGCAAGCTTCGTACGCTGCAG 3’

**Table S1.** Plasmids generated in this study

| Plasmid name | Overexpressed gene(s) | Promoter used for overexpression | Plasmid origin of replication | Selectable marker | Reference |
| --- | --- | --- | --- | --- | --- |
| pRSII416-SsXKS | *XKS* from *S. stipitis* | *TEF1* | CEN6/ARS4 | Ura3 | This study |
| pRSII416-SsXR | *XYL1* from *S. stipitis* | *TEF1* | CEN6/ARS4 | Ura3 | This study |
| pRSII416-SsXDH | *XYL2* from *S. stipitis* | *TEF1* | CEN6/ARS4 | Ura3 | This study |
| pRSII416-ScHXT7mut | *HXT7F79S* from *S. cerevisiae* | *TEF1* | CEN6/ARS4 | Ura3 | This study |
| pRSII416-LlkivD-T2A-ScADH7 | *LlkivD*_mito from *Lactococcus lactis*, *ADH7* from *S. cerevisiae* | *TEF1* | CEN6/ARS4 | Ura3 | This study |
| pRSII416-ScIlv2-T2A-ScIlv5-T2A-ScIlv3 | *Ilv2*, *Ilv5* and *Ilv3* from *S. cerevisiae* | *TEF1* | CEN6/ARS4 | Ura3 | This study |
| pRSII426-SsXKS | *XKS* from *S. stipitis* | *TEF1* | 2μm | Ura3 | This study |
| pRSII426-SsXR | *XYL1* from *S. stipitis* | *TEF1* | 2μm | Ura3 | This study |
| pRSII426-SsXDH | *XYL2* from *S. stipitis* | *TEF1* | 2μm | Ura3 | This study |
| pRSII426-ScHXT7mut | *HXT7F79S* from *S. cerevisiae* | *TEF1* | 2μm | Ura3 | This study |
| pRSII426-LlkivD-T2A-ScADH7 | *LlkivD*_mito from *Lactococcus lactis*, *ADH7* from *S. cerevisiae* | *TEF1* | 2μm | Ura3 | This study |
| pRSII426-ScIlv2-T2A-ScIlv5-T2A-ScIlv3 | *Ilv2*, *Ilv5* and *Ilv3* from *S. cerevisiae* | *TEF1* | 2μm | Ura3 | This study |
